# Undifferentiated embryonal sarcoma of the liver mimicking acute appendicitis. Case report and review of the literature

**DOI:** 10.1186/1477-7819-4-9

**Published:** 2006-02-20

**Authors:** Timothy Sakellaridis, Ioannis Panagiotou, Themistoclis Georgantas, George Micros, Dimitra Rontogianni, Christos Antiochos

**Affiliations:** 1 1^*st *^Surgical Department, 401 Army Force General Hospital of Athens, Greece; 2 Pathologic Department, Evagelismos, General Hospital of Athens, Greece

## Abstract

**Background:**

Undifferentiated embryonal sarcoma (UES) of liver is a rare malignant neoplasm, which affects mostly the pediatric population accounting for 13% of pediatric hepatic malignancies, a few cases has been reported in adults.

**Case presentation:**

We report a case of undifferentiated embryonal sarcoma of the liver in a 20-year-old Caucasian male. The patient was referred to us for further investigation after a laparotomy in a district hospital for spontaneous abdominal hemorrhage, which was due to a liver mass. After a through evaluation with computed tomography scan and magnetic resonance imaging of the liver and taking into consideration the previous history of the patient, it was decided to surgically explore the patient. Resection of I–IV and VIII hepatic lobe. Patient developed disseminated intravascular coagulation one day after the surgery and died the next day.

**Conclusion:**

It is a rare, highly malignant hepatic neoplasm, affecting almost exclusively the pediatric population. The prognosis is poor but recent evidence has shown that long-term survival is possible after complete surgical resection with or without postoperative chemotherapy.

## Background

Stocker *et al*, named undifferentiated embryonal sarcoma of the liver, as an entity in 1978 on the basis of an Armed Forces Institute of Pathology (AFIP) series [1]. It is a rare, highly malignant hepatic neoplasm, affecting almost exclusively the pediatric population. The prognosis is poor but recent evidence has shown that long-term survival is possible after complete surgical resection with or without postoperative chemotherapy. We report our experience treating a 20-year-old Caucasian male with undifferentiated embryonal sarcoma of the liver.

## Case presentation

**Figure 1:**
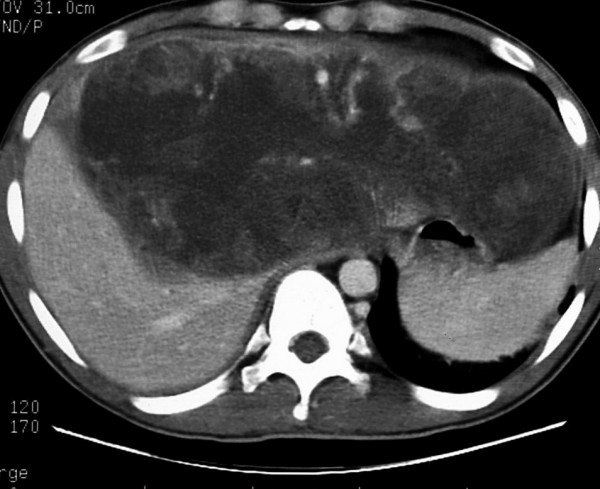
Computed tomography (CT) of the abdomen demonstrating hepatomegaly and a large hypodense lesion of the liver with multicystic appearance with septations and solid portions.

**Figure 2:**
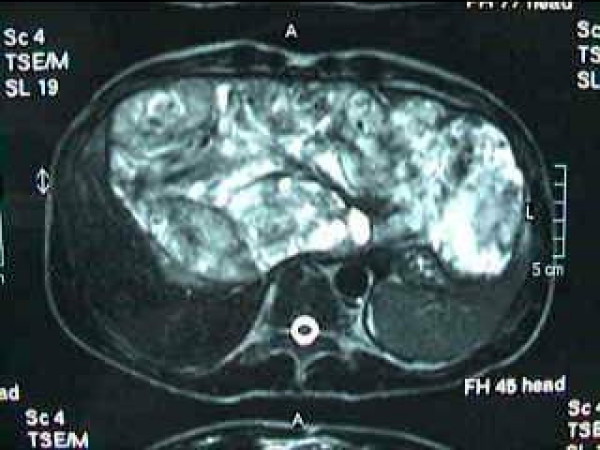
Magnetic Resonance Imaging (MRI) in T2-weighted and Short Time Inversion Recovery (STIR) sequences revealing hyperdense areas with intermixed hypodense septa.

**Figure 3:**
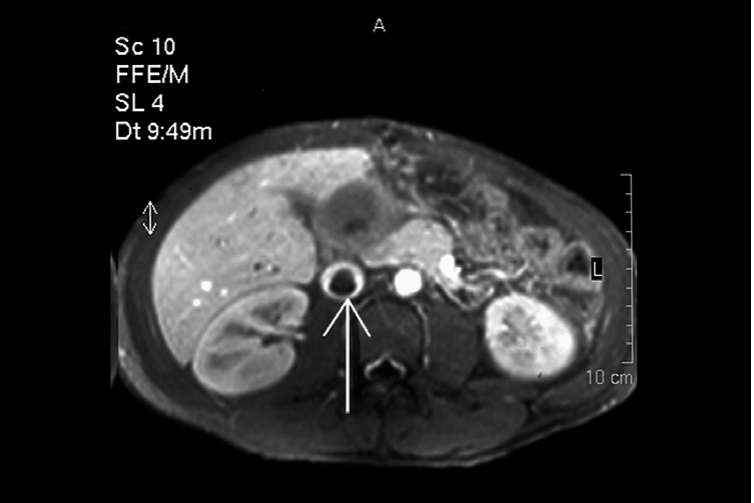
MRI revealing a thrombus in the inferior vena cava (the arrow shows the thrombus)].

**Figure 4:**
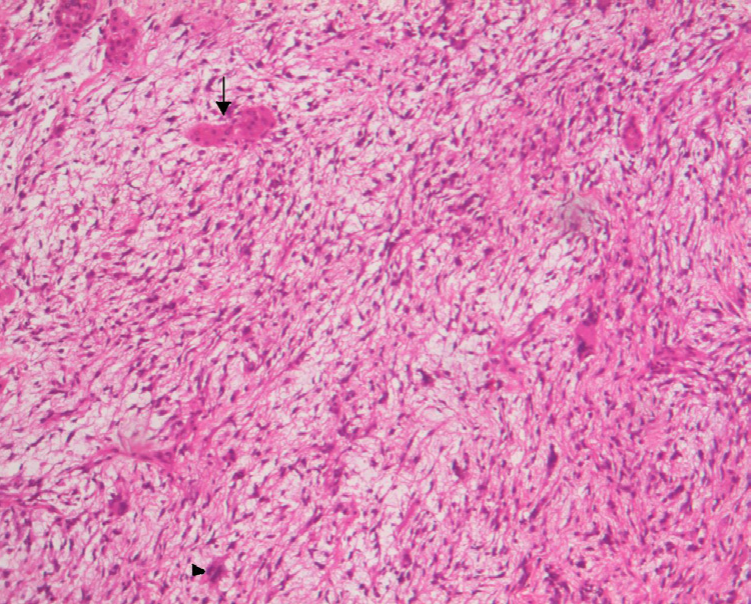
Microphotograph of malignant sarcomatous tissue with spares giant neoplastic cells (head arrow) and residual hepatocytes (arrow) (H-E stain, × 100).

**Figure 5:**
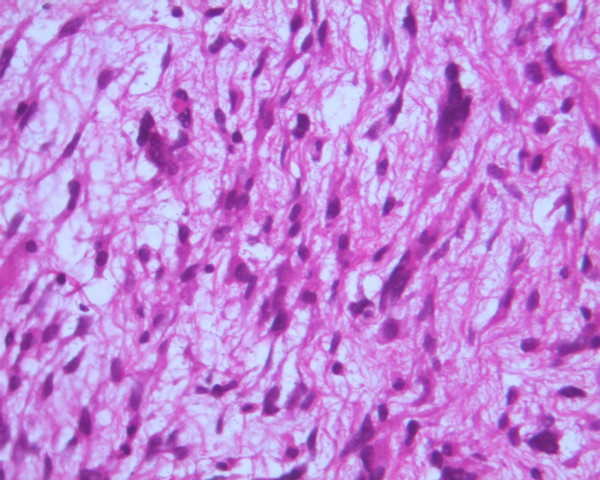
Sarcomatous tissue with severe atypia of the neoplastic cells and focal presence of giant cells partially with myoblastic characteristics (H-E stain, × 400).

A 20-years-old Caucasian male, presented to a district hospital with acute right lower quadrant pain from six hours before. Physical examination revealed abdominal pain, localized right lower quadrant abdominal tenderness, body temperature slightly elevated (37.7°C), blood pressure 120/90 mmHg and heart rate 110/min. Blood tests showed leukocyte count at 12600/μL, the differential white account showing 75% neutrophils and hematocrit 35.9%. Abdominal and chest radiographs were normal. The primary diagnosis was acute appendicitis and appendectomy was decided. Entering the peritoneal cavity from a McBurney incision, the surgeon found large amount of blood and the surgery was transformed to exploratory laparotomy through a midline incision. The findings were enormous hepatomegaly and bleeding due to spontaneous rupture of the IV lobe of the liver. Active bleeding was managed with direct suture of identifiable vessels and absorbable gauze mesh. Postoperative course was uneventful, except of a continuous elevated body temperature (up to 38.5°C). The patient, five days after surgery, was referred to us in order to undergo further examinations for the nature of the liver abnormality. His medical history was unremarkable. He was previously in good health. His family and past history were not contributory. The patient weighted 65 Kg with 1.75 m height. He was in his right senses and well nourished. Yellow skin, icteric sclera, spider naevi and palmar erythema were not founded. Cardiopulmonary examination was unremarkable. Chest radiograph revealed no pulmonary pathology. The abdomen was swollen with the liver palpable. Laboratory tests showed liver function tests normal (before the initial surgery however, after the surgery SGOT, SGPT, LDH were altered three times the normal range) hepatitis markers of HAV, HBV, HCV, and HIV were negative and alpha-fetoprotein (AFP) was 35 ng/ml. The patient underwent computed tomography (CT) of the abdomen that showed hepatomegaly and a large hypodense lesion of the liver with multicystic appearance with septations and solid portions (Figure 1). Magnetic Resonance Imaging (MRI) in T2-weighted and Short Time Inversion Recovery (STIR) sequences revealing hyperdense areas with intermixed hypodense septa (Figure 2), and a thrombus in the inferior vena cava (Figure 3). The differential diagnosis by the CT and MRI findings was giant hemangioma or cystic malignant lesion. Due to the size of the lesion and the previous history of spontaneous bleeding, it was decided that the patient must undergo surgery. In order to prevent pulmonary embolism, an inferior vena cava filter was inserted and fixated just below the renal veins. Two days later the patient underwent surgery. Primarily, thrombectomy and reconstruction of the inferior vena cava with removal of the filter was performed, followed by hepatectomy of I–IV and VIII lobe of the liver. Intraoperative biopsy demonstrated malignancy or sarcoma of the liver. Frozen section on the remaining liver was performed and showed normal liver tissue. The patient after surgery presented with life threatening bleeding and protean coagulation abnormalities, symptoms of disseminated intravascular coagulation, due to massive blood transfusion. The patient died the second postoperative day due to uncontrolled bleeding caused of the developing of disseminated intravascular coagulation (DIC). Pathologic review of the specimen revealed a 1580 gm left and part of right hepatic lobe, with a 30 × 14 × 9 cm tumor mass and the histological examination showed malignant sarcomatous tissue with spares giant neoplastic cells (head arrow) and residual hepatocytes (arrow) suggestive of undifferentiated embryonal sarcoma (Figure 4). Sarcomatous tissue with severe atypia of the neoplastic cells and focal presence of giant cells partially with myoblastic characteristics was also present (Figure 5). Residual hepatocytes stained positive in immunohistochemical stain for cytoceratinin 8–18, and showed infiltration by the sarcomatous tissue (DAB technique).

## Discussion

Undifferentiated embryonal sarcoma (UES) of the liver was first described as a clinicopathologic entity in 1978. Before that, the nomenclature was variable and included mesenchymoma, primary sarcoma of the liver, fibromyxosarcoma and malignant mesenchymoma [2].

After neuroblastoma and Wilm's tumor, primary hepatic neoplasms are the third most common solid malignant tumors found in children accounting for 2% of the total of childhood cancers. Although rare, malignant mesenchymal hepatic tumors are the fourth most common malignant tumors of the liver in the children, with hepatoblastoma and hepatocellular carcinoma accounting for the majority of primary hepatic malignancies in children [2,3].

The UES of the liver is typically diagnosed after 6 years of age with a decline in incidence after 10 years of age and 50% of the patients were between the above ages in the longest reported series [4]. In children, there is a slight male predominance (1.0:0.65) [2]. In 1978, 31 patients were reported with embryonal sarcoma with a median survival time of less than 1 year [1]. A recent review of published cases revealed a better outlook with 37.5% of patients alive without disease for an average of 37.5 months [5]. Two further patients are reported alive and free of disease more than 5 years after complete surgical excision of tumor and chemotherapy [5].

The UES of the liver is a rare tumor that most often presents in childhood but infrequently in adults. Less than 60 cases of UESL in adults have been reported in the pertinent literature [2,6-9].

There are no specific clinical features. Tumor related symptoms might be regarded as abdominal mass with or without upper abdominal pain or swelling. Fever is probably related to the hemorrhage and necrosis found in the majority of these tumors, as happened in our patient. Of possible significance is the absence of jaundice. Rupture into the tumor or into the peritoneal cavity due to rapid growth of the tumor is not uncommon [10]. Review of the literature did not reveal a case of undifferentiated embryonal sarcoma mimicking acute appendicitis. Different from primary carcinoma of the liver, UESL has no relation to hepatitis or liver cirrhosis, no disturbance of hepatic function. Laboratory studies are non-specific, and the α-fetoprotein is not increased [1,2,7].

Radiographs of the abdomen are usually normal. The lesion can be detected by ultrasound, CT and MRI. MRI localizes the lesion more accurately than the other methods, with good resectability correlation. It also can detect vascular invasion, biliary obstruction and hilar adenopathies [11].

UES of the liver is a neoplasm with primitive mesenchymal phenotype. Tumor size often exceeds 10 cm and can be as large as 30 cm. Macroscopic examination shows a single, well-demarcated, soft, globular mass that frequently has cystic, gelatinous, hemorrhagic and necrotic foci. Microscopic examination reveals a pseudocapsule surrounding a neoplasm, composed predominately of spindle, oval, or stellate cells with ill-defined cell borders. The tumor cells are embedded in an abundant myxoid stroma that contains many thin-walled veins. The bile duct-like structures occasionally appear hyperplastic or reactive, although they, too, may show degenerative changes [1]. Immunohistochemical studies have indicated variable immunoreactivity with antibodies to desmin, muscle-specific actin, and cytokeratin, but not myoglobin [3]. Vimentin and the "histiocytic" determinants (alpha-1-antitrypsin and alpha-1-antichymotrypsin) have been the only consistent immunohistochemical markers expressed by this tumor. However, it is known that the latter two markers are not specific for histiocytes, but are expressed in a range of tissue types including epithelium [4]. In our case the immunohistochemical studies revealed expression to vimentin, desmin, but not to muscle-specific actin, S-100-protein and cytokeratin.

Evaluable treatments included surgery, hepatic arterial ligation, hepatic transplantation, and combinations of surgery and/or chemotherapy and radiation therapy. Radical resection of the tumor is the optimal treatment of choice [12,13]. According to McFadden et al, combination chemotherapy and radiation therapy, followed by elective resection, should be considered for all adult patients with UES. Similar protocols are reasonable for pediatric patients too [2,14].

The prognosis for UESL has been poor to until recently and majority of the patients died of tumor recurrence or metastasis within two years after the initial operation [1]. The major impediments in achieving long-term, disease-free intervals, is local recurrence in the upper abdomen and distant metastases [4]. Metastases to lung, pleura and peritoneum are common; invasion of the vena cava with extension into the right atrium rarely occurs [3]. Recent researchers have shown that pre- and/or post-operative systemic chemotherapy (with cisplatin, andriamycin, cyclophosphamide) and/or radiotherapy, when necessary, can remarkably improve patient's survival [15,16]. Because the tumor does not produce any characteristic serum markers to permit monitoring of subclinical recurrences, the second-look laparotomy after the completion of chemotherapy should be considered [4]. Once there is an evidence of recurrence, resection of the tumor wherever feasible should be performed [7].

## Competing interests

The author(s) declare that they have no competing interests.

## Authors' contributions

All authors contributed equally in preparation of the manuscript.

**Figure 6:**
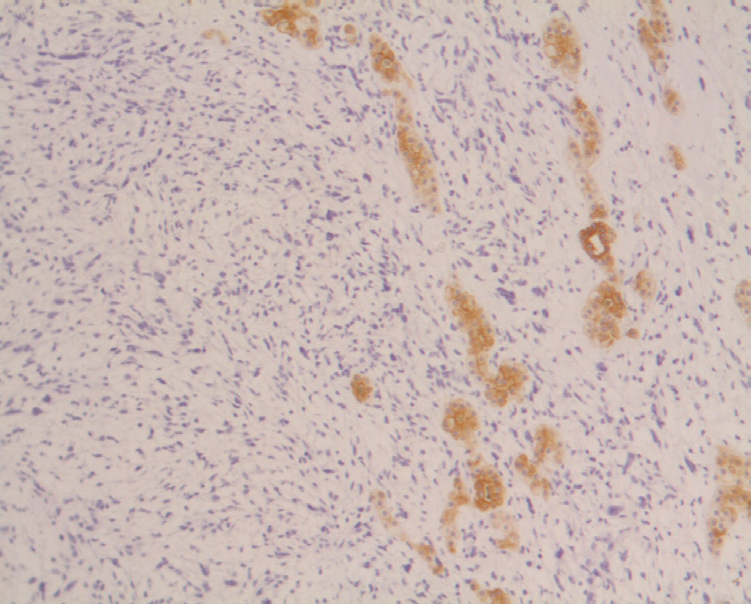
Residual hepatocytes positive in immunohistochemical stain for cytoceratinin 8–18, infiltrated by the sarcomatous tissue (DAB technique, ck 8–18, ×100)].
